# Cations substitution tuning phase stability in hybrid perovskite single crystals by strain relaxation

**DOI:** 10.1039/c7ra12521f

**Published:** 2018-01-15

**Authors:** C. Wu, K. Chen, D. Y. Guo, S. L. Wang, P. G. Li

**Affiliations:** Department of Physics, Center for Optoelectronics Materials and Devices, Zhejiang Sci-Tech University Hangzhou 310018 China slwang@zstu.edu.cn pgli@zstu.edu.cn

## Abstract

Methylammonium (MA) and formamidinium (FA) are two typical A site cations in lead halide perovskites. Instability of synthesised crystals will degrade the properties of the photoelectrical device constructed by such perovskites. MAPbI_3_ and FAPbI_3_ in cubic crystal structure have been demonstrated to be the most stable at room temperature. Herein we synthesised MA(EA)PbI_3_ and FA(MA)PbI_3_ single crystals using an inverse-temperature crystallization strategy by partially substituting the methylammonium (MA) with ethylammonium (EA) and the formamidinium (FA) with methylammonium (MA) respectively. The XRD results show that both crystal structures are cubic, which means organic incorporation can stabilize the crystal structure of lead halide perovskites. The lattice distortion decrease and strain relaxation in single crystals were considered to be the reason leading to higher stability. The single crystals of MA(EA)PbI_3_ and FA(MA)PbI_3_ with low trap state density exhibit excellent light-absorbing properties, indicating their potential applications in photoelectric devices.

## Abstract 2

Cations size induced phase tuning in hybrid perovskite single crystals: interplay of lattice distortion and strain relaxation.

## Graphical abstract

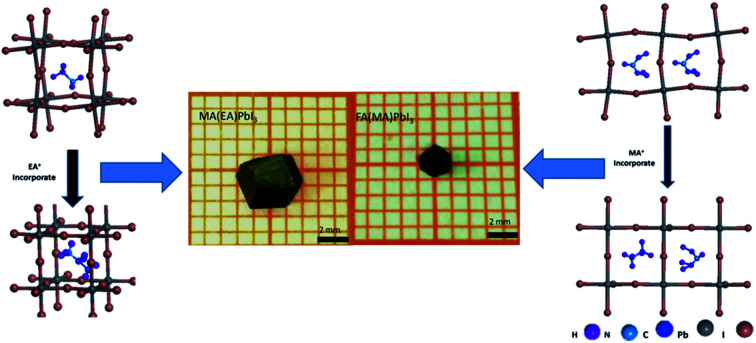

## Introduction

Organic-lead trihalide hybrid perovskites have been widely investigated for solar cells,^1^ photodetectors,^2^ lasing,^3^ light-emitting diodes,^4^ and hydrogen production,^5^ owing to their superior characteristics including direct bandgap, highly balanced hole and electron mobility, strong absorption coefficient and long carrier lifetime *etc.*^6^ As is well known, the perovskite structure of ABX_3_ (where A is an organic cation, B is a metal cation, and X is a halide anion) consists of a three-dimensional array of [BX_6_] octahedra with a cation occupying the 12-coordinated cubo-octahedral cavities of the 3D network. In the ABX_3_ perovskite structure, the size of precursor ions needs to follow one principal, which can be expressed as 
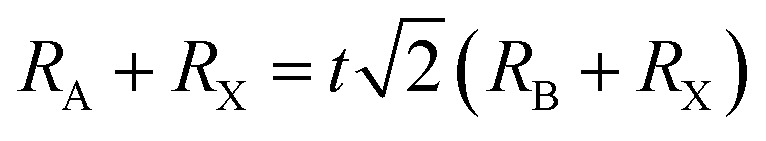
, where *R*_A_, *R*_B_ and *R*_X_ are the ionic radii of the corresponding ions. When the tolerance factor *t* of the perovskite is between 0.8 to 1, a stable three-dimensional (3D) crystal structure can be obtained. While maintaining a high-symmetry cubic structure, the value of *t* should be close to 1.^7^ Currently, the most-studied lead halide perovskites usually have formamidinium (FA) or methylammonium (MA) at the A site.

In fact, there are three types of MAPbI_3_ crystals, orthorhombic, tetragonal and cubic structures.^8^ Two phase transitions occur at 162.2 K and 327.4 K for orthorhombic–tetragonal and tetragonal–cubic transitions, respectively.^9^ The phase transition of MAPbI_3_ from cubic to tetragonal phases at 327.4 K may cause undesired lattice distortion and strain which is harmful to photoelectric devices.^10^ Compared with MAPbI_3_, FAPbI_3_ perovskite demonstrates better thermal stability and even better photoelectric property. However, FAPbI_3_ suffers from the well-known spontaneous phase transition from the desired cubic phase (α phase) black perovskite to δ-phase yellow non-perovskite at room temperature.^11^ This phase transition is the main obstacle for high efficiency and long-term stability of FAPbI_3_-based optelectric devices. Therefore, an urgent assignment is engineering and synthesizing of cubic MAPbI_3_ and FAPbI_3_ crystals which can stability exist at room temperature.

Zhu *et al.*^12^ reported the incorporation of cations with smaller effective radius can adjust the tolerance factor and relax the crystal strain of FA-based perovskites. Peng *et al.*^13^ studied the incorporation of cations with bigger effective radius to obtain cubic phase perovskite. M. T. Weller and O. J. Weber's research group have investigated the routes and kinetics of degradation of thin films of methylammonium (MA)/formamidinium (FA) lead iodide perovskites (FA_*x*_MA_1−*x*_PbI_3_).^14^ Meantime, they focused on growth of MA/FA system perovskite crystals and exploration of the phase transition mechanism.^15^ Herein, we synthesised cubic phase MA(EA)PbI_3_ and FA(MA)PbI_3_ single crystals by an inverse-temperature crystallization strategy, and investigated their thermodynamic and electronic property.

## Experimental section

### Materials

PbI_2_ (99.99%) was obtained from Macklin. MAI (>98%, 2-time purification), as well as EAI (>99%, 4-time purification) and FAI (>99%, 4-time purification) were acquired from Chengdu Technology Co. Ltd. The solvent γ-butyrolactone (GBL, 99%) was also obtained from Macklin. All materials were used as received.

### Growth of the mixed-perovskite crystals

Perovskite crystals were grown by a reported method of inverse temperature crystallization.^16^ Briefly, 1 M PbI_2_ and 1 M CH_3_NH_3_I (MAI) were dissolved in 2 ml γ-GBL at 40 °C, and stirred until the solution becomes clear. The solution was then kept at 90 °C for about 12 h to allow for pure MAPbI_3_ crystal growth. For the FA^+^ cation mixed with MAPbI_3_, 0.922 g PbI_2_, 0.159 g MAI, and 0.172 g HC(NH_2_)_2_I(FAI) were dissolved in 2 ml GBL solution under the same conditions outlined above, to facilitate mixed MAPbI_3_ crystal growth. This same method has been used for the incorporation of EA cations.

### Characterization of the mixed-perovskite crystals

X-ray diffraction (XRD) data from single crystals were collected by a Bruker D8-Advance, using Cu Kα radiation. Thermogravimetric analysis (TGA) was performed on a TGA analyzer (PYRTS 1). Differential scanning calorimetry (DSC) analysis was carried out by using Q2000 to test phase transition. Photoluminescence (PL) measurements of bulk crystals were performed with a Renishaw inVia Raman Microscope using a 532 nm laser as excitation source. The ^1^H Nuclear Magnetic Resonance (NMR) spectra were recorded in dimethyl sulfoxide (DMSO) using a Bruker Advance 300 spectrometer. UV-vis diffuse reflectance spectroscopy was measured using a UV-vis spectrophotometer (U-3900). *V*–*I* characteristics were tested using a Keithley 2400 instrument.

## Result and discussion

**Fig. 1:**
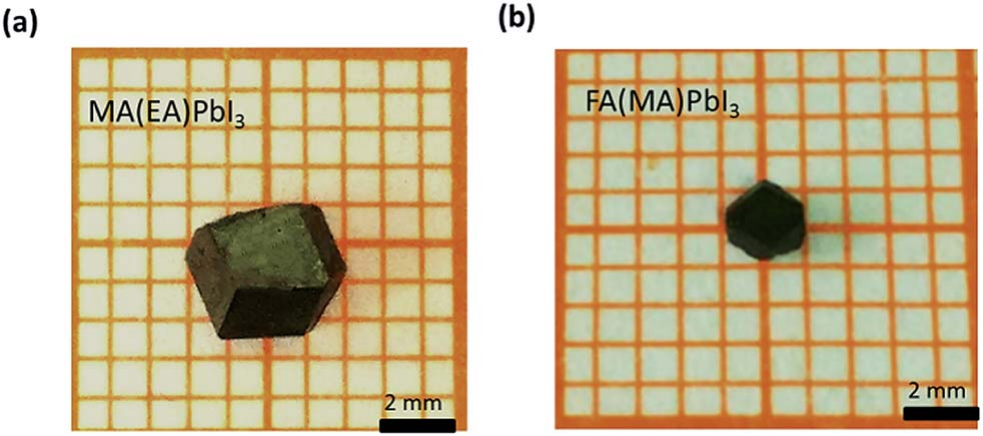
Images of cubic-phase single crystals (a) MA(EA)PbI_3_ and (b) FA(MA)PbI_3_.

**Fig. 2:**
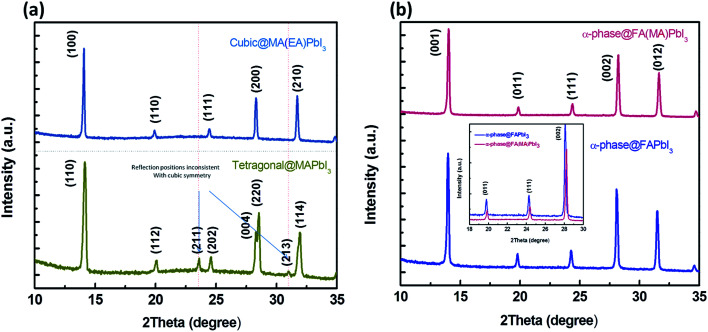
(a) Powder XRD patterns of the cubic MA(EA)PbI_3_ and the XRD of tetragonal crystal is also presented. (b) Powder XRD of α-FAPbI_3_ and α-FA(MA)PbI_3_.

**Fig. 3:**
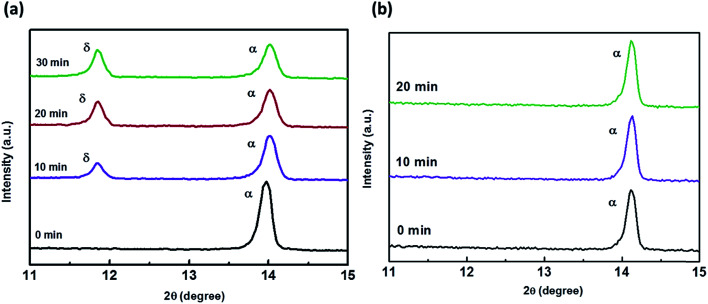
Time-dependent XRD of FAPbI_3_ (without MA) and FA(MA)PbI_3_ (with MA).

**Fig. 4:**
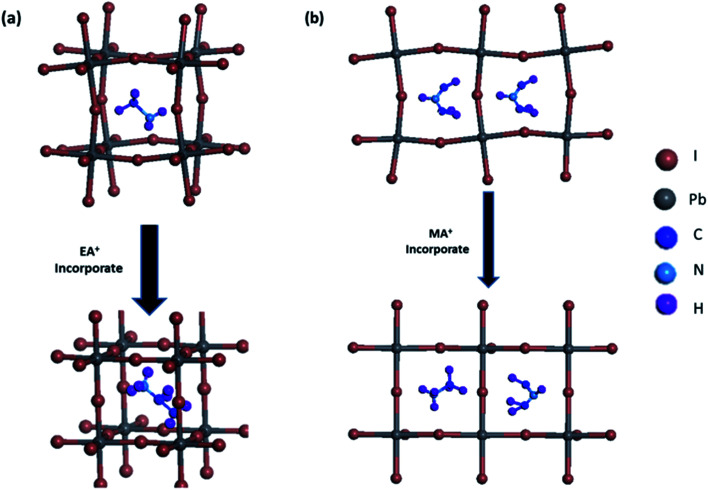
Schematic representation of organic cation incorporated (a) MA(EA)PbI_3_; (b) FA(MA)PbI_3_.

MA(EA)PbI_3_ precursors solutions were prepared using MAI and EAI with a molar ratio of 1 : 3 while FA(MA)PbI_3_ precursors solutions were prepared using MAI and FAI with a molar ratio of 1 : 1. After growing for about 12 h, the single crystal sizes of 4 mm × 3 mm × 1 mm and 2 mm × 2 mm × 1 mm are finally obtained for MA(EA)PbI_3_ and FA(MA)PbI_3_ respectively, as shown in Fig. 1. XRD patterns of the perovskite crystals are shown in Fig. 2. It shows that the main peaks for MA(EA)PbI_3_ is at 2*θ* = 14.1°, 28.3° and 31.7°. The diffraction pattern of the tetragonal MAPbI_3_ crystal is also shown in Fig. 2a for comparing. The reported calculated and experimental data of MAPbI_3_ show that the X-ray peaks (211) and (213) were used to differentiate tetragonal and cubic phase.^17^ No (211) reflection at 2*θ* = 23.5° was observed, which proves that MA(EA)PbI_3_ has a cubic structure. Previously report the sizes of the MA^+^ (2.03 Å) and EA^+^ (2.42 Å) were calculated.^18^ We speculate mixed of different size organic cations in perovskite caused lattice dilation, altered Pb–I–Pb bond angle and finally increased the crystal symmetry. When the A site is occupied by big size organic cation such as EA^+^, the lead halide perovskite will become a two-dimensional (2D) layer structure. This is due to the large ionic radius of EA^+^ resulting in a tolerance factor out of the empirical range for a stable 3D perovskite structure. We propose that MA(EA)PbI_3_ could show a stable 3D PbI_6_ octahedral framework owing to two lattice-distortion factors: (1) the small radius of MA^+^ causes lattice contraction; (2) the large radius of EA^+^ causes lattice dilation. This cell dilation adjusts the tolerance factor toward 1, favourable to stabilize the cubic perovskite. The diffraction pattern of FA(MA)PbI_3_ in a good agreement with the recently reported cubic phase FAPbI_3_ which were shown in Fig. 2b. Strain in the (111) plane of the cubic phase FAPbI_3_ is a driving force for its easy phase transition into the δ-phase, where the (111) plane acts as the nucleation site for the (0001) δ-phase.^19^ When small organic cation MA^+^ incorporated, the strain in (111) was relaxed. As shown in the inset of Fig. 2b, the diffraction peak positions shift to higher angles when MA^+^ cation incorporates, indicating the decrease of the lattice plane space. The FWHM for cubic phase FAPbI_3_ is 0.187° while for FA(MA)PbI_3_ is 0.177°. The sharpening of the peak indicates the relaxation of strain.^20^Fig. 3 shows the time-dependent XRD measurements of FAPbI_3_ and FA(MA)PbI_3_ crystals. In the air at room temperature, FAPbI_3_ exhibits a phase transition while FA(MA)PbI_3_ does not. Spontaneous phase transition of FAPbI_3_ from cubic phase to non-perovskite phase was prevented when MA^+^ incorporated. The result is consistent with the reported literature.^15^ Schematic representation of the incorporation of organic cations was shown in Fig. 4. Cell parameters of MA(EA)PbI_3_ and FA(MA)PbI_3_ crystals were shown in Table 1.

**Table 1:** Cell parameters of MA(EA)PbI_3_ and FA(MA)PbI_3_

	Phase	Space-group	Cell-parameters	Cell volume
MA(EA)PbI_3_	Cubic	*Pm*3̄*m*	6.309 Å	251.120 Å^3^
FA(MA)PbI_3_	Cubic	*Pm*3̄*m*	6.329 Å	253.516 Å^3^

**Fig. 5:**
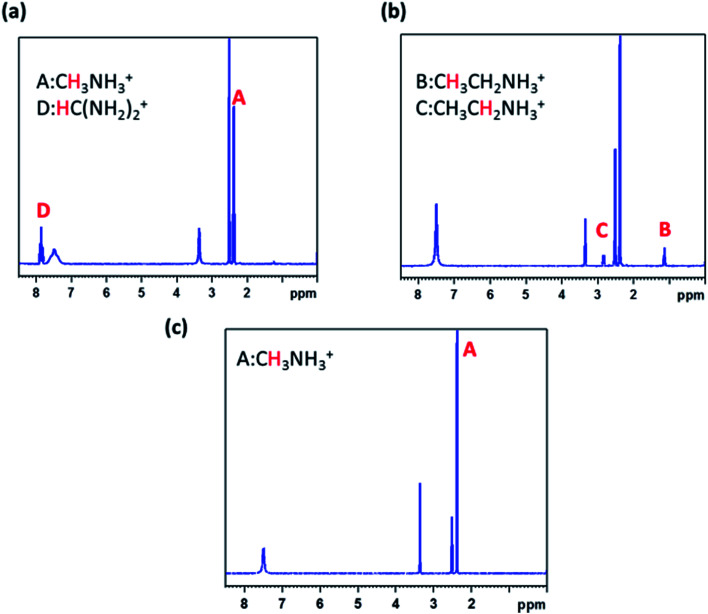
^1^H NMR spectra of the cubic samples, (a) FA(MA)PbI_3_; (b) MA(EA)PbI_3_; (c) MAPbI_3_.

Unfortunately, the organic components show much lower diffraction intensity and intense rotating motion, we could not resolve their distributions.^18^ To further confirm the composition, we used solution-phase ^1^H NMR spectroscopy. The species corresponding to the 1H peaks are listed. Compared with tetragonal MAPbI_3_ sample, we can identify B, C 1H species in MA(EA)PbI_3_ and D 1H species in FA(MA)PbI_3_. It means that EA and MA had incorporated in MAPbI_3_ and FAPbI_3_, respectively. From ^1^H NMR spectroscopy of MA(EA)PbI_3_, integration of B and C peaks shows a B/C ratio of 1.00 : 0.66 which is consistent with the proton population ratio –CH_3_/–CH_2_– in EA^+^. The B/A ratio is 1 : 6 which indicates an EA/MA ratio of 1 : 6. While from ^1^H NMR spectroscopy of FA(MA)PbI_3_, A/D ratio indicates a FA/MA ratio of 1 : 1 (Fig. 5).

**Fig. 6:**
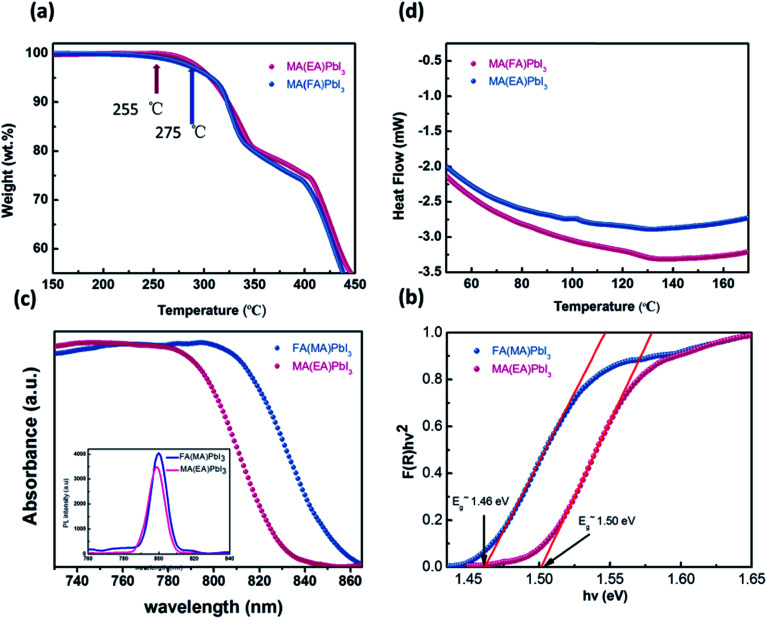
(a) TGA curves cubic phase crystals; (b) DSC heating curve of samples; (c) UV-vis diffuse reflectance spectrum and photoluminescence (PL) properties; (d) bandgap determination.

To examine the thermal properties of perovskite MA(EA)PbI_3_ and FA(MA)PbI_3_ thermogravimetric analysis (TGA) was measured from room temperature to 500 °C under nitrogen flow. As shown in Fig. 6a, the decomposition temperature of MA(EA)PbI_3_ is about 255 °C, which is slightly higher than that of MAPbI_3_ single crystal (240 °C). While FA(MA)PbI_3_ decomposed at 275 °C, which is smaller than FAPbI_3_ (300 °C). It should be noted that this decomposition, by sequential loss of HI followed by organic part, only occurs when the organic species are incorporated into the perovskite structure.^14^ The identical thermal behavior was also observed from differential scanning calorimetry (DSC) in Fig. 6b. The thermal stability is related to the probability of HI formation, which is directly related to the acidity of the organic cation.^21^ The stronger the acidic character of the cation, the higher the chance that the organic cation can be deprotonated to yield HI. Since the FA cation is less acidic than MA and EA organic species, it is natural that the thermal decomposition is difficult in FA-incorporated perovskites. The pure FAPbI_3_ exhibits a peak at 156 °C, indicating a phase transition of FAPbI_3_ from the yellow δ phase to the perovskite structure at this temperature. MAPbI_3_ shows a peak at 57 °C which indicates a phase transition of tetragonal–cubic transition. Neither of the mixed perovskites shows any peaks, indicating that these mixed perovskites are stable over the investigated temperature range. UV-vis diffuse reflectance of perovskites were characterized by an UV-vis diffuse reflectance spectrometer to confirm the band gap energy of single crystals. Reflectance spectra for the single crystals as a function of wavelength in the range of 740–860 nm are presented in Fig. 6c. A further analysis of optical spectra can be performed to calculate band gap energy. The Kubelka–Munk equation at any wavelength is1
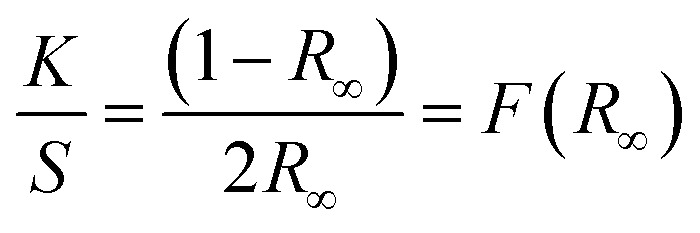
where *S* and *K* are scattering absorption and coefficients, respectively. *F*(*R*_∞_) is called the Kubelka–Munk function. The band gap *E*_g_ and the absorption coefficient *α* of a direct band gap semiconductor are related through the well-known equation^22,23^2
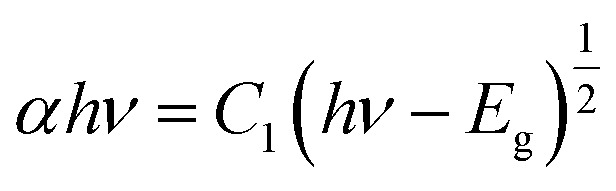
where *α* is linear absorption coefficient of the material, *hν* is the photon energy, and *C*_1_ is a proportionality constant. When the material scatters in a perfectly diffuse manner, the Kubelka–Munk absorption coefficient *S* is constant with respect to wavelength, and using the remission function in eqn (2), we obtain the expression:^24^

3 [*F*(*R*_∞_)*hν*]^2^ = *C*_2_(*hν* − *E*_g_)

Therefore, obtaining *F*(*R*_∞_) from eqn (1) and plotting [*F*(*R*_∞_)*hν*]^2^ against *hν*, the band gap energy, *E*_g_, can be obtained easily, and are shown in Fig. 6d.

The optical bandgap of MA(EA)PbI_3_ is determined to be about 1.49 eV which is similar to MAPbI_3_. While the value of FA(MA)PbI_3_ is about 1.43 eV, that is smaller than MAPbI_3_. Fig. 6c also shows the photoluminescence (PL) spectra of the perovskite. MA(EA)PbI_3_ and FA(MA)PbI_3_ exhibit narrow PL peaks at 709 nm and 800 nm, respectively. PL measurement shows the light emission peak position of the single crystal is very close to the absorption onset, indicating low trap state density. From the UV-vis spectra and PL spectra, both MA(EA)PbI_3_ and FA(MA)PbI_3_ have superior light-absorbing capability which holds potential to be a suitable photoelectric material.

**Fig. 7:**
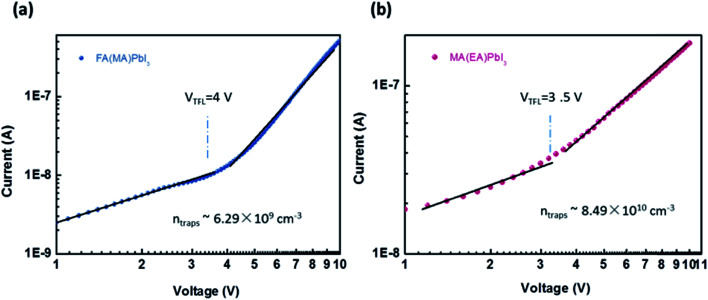
(a) and (b) Current–voltage curve for a hole only MA(EA)PbI_3_ device and hole only FA(MA)PbI_3_ device, respectively.

The trap density (*n*_trap_) in cubic phase single crystals was investigated by using dark *I*–*V* technique to characterize fabricated hole-only device. When the applied voltage is lower than the kink-point voltage, the current *I* increase linearly with applied voltage *V*, demonstrating an ohmic response between the electrode and the perovskite for the hole-only device. As the applied voltage exceeds the kink-point voltage the current *I* exhibit a quick non-linear increase, indicating that the trap states are fully filled by the injected carriers. The applied voltage at the kink point is defined as the trap-filled limit voltage (*V*_TFL_), which is determined by the trap state density:^19^4
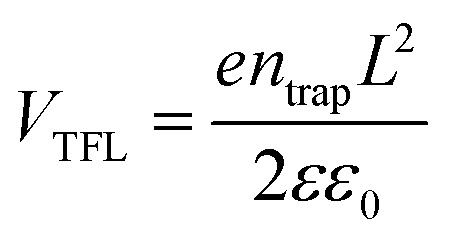
where *L* is the thickness of the perovskite single crystals, *ε* is the relative dielectric constant and *ε*_0_ is the vacuum permittivity. Hence the trap *n*_trap_ can be calculated using eqn (1). Based on Fig. 7a and b, the corresponding hole trap density is 8.49 × 10^10^ cm^−3^ for MA(EA)PbI_3_ and 6.29 × 10^9^ cm^−3^ for FA(MA)PbI_3_. The values are declined by most one order magnitude of these found in MAPbI_3_,^25^ demonstrating the high quality of these new materials.

## Conclusions

In summary, we report a route to synthesis cubic phase crystal MA(EA)PbI_3_ and FA(MA)PbI_3_ by using inverse temperature reactive crystallization process. Big size organic cation EA^+^ incorporated into MAPbI_3_ and small size MA^+^ mixed with FA(MA)PbI_3_ could obtain stable cubic single crystal *via* altering the PbI_6_ octahedral cage and relaxed strain. The large radius of EA^+^ causes lattice dilation and adjusts the tolerance factor toward 1, favourable to stabilize the cubic perovskite. MA^+^ cation incorporated has reduced the lattice volume and relaxed the strain in lattice and thereby prevent the phase transition from the cubic phase to δ-phase. Both MA(EA)PbI_3_ and FA(MA)PbI_3_ single crystals show remarkable thermal stability with no endothermic peak at range 50–170 °C. Direct dark *I*–*V* measurement of cubic phase crystals indicates low trap state density. Both MA(EA)PbI_3_ and FA(MA)PbI_3_ have superior light-absorbing capability which holds potential to be a suitable photoelectric material.

## Conflicts of interest

There are no conflicts to declare.

## Supplementary Material
